# Comprehensive pan-cancer analysis of 33 human cancers reveals immunotherapeutic value of focal adhesion tyrosine kinase

**DOI:** 10.1097/MD.0000000000037362

**Published:** 2024-03-22

**Authors:** Yujing Shi, Mengyang Ju, Yumeng Zhang, Liang Liang, Xinchen Sun, Xiaoke Di

**Affiliations:** a Department of Oncology, The People’s Hospital of Jurong City, Jurong Hospital Affiliated to Jiangsu University, Zhenjiang, China; b Department of Radiation Oncology, Osaka University Graduate School of Medicine, Osaka, Japan; c Department of Radiation Center, Shanghai First Maternity and Infant Hospital. School of Medicine, Tongji University, Shanghai, China; d Department of Radiotherapy, Jiangsu Provincial People’s Hospital, Nanjing, China.

**Keywords:** FAK/PTK2, immune checkpoint inhibitors, immunotherapeutic response, tumor microenvironment

## Abstract

The immune environment in tumors is the key factor affecting the survival and immunotherapeutic response of patients. This research aimed to explore the underlying association between focal adhesion tyrosine kinase (FAK/PTK2) and cancer immunotherapy in 33 human cancers. Gene expression data and clinical features of 33 cancers were retrieved from the Cancer Genome Atlas Database. The immunotherapy cohorts included GSE67501, GSE78220, and IMVIGOR210, which were derived from the comprehensive gene expression database or from previous studies. Clinical parameters including patient age, gender, survival rate, and tumor stage were analyzed to evaluate the prognostic value of FAK/PTK2. FAK/PTK2 activity was detected by single-sample gene set enrichment analysis and used to compare the difference between FAK/PTK2 transcriptome and protein expression levels. To better understand the role of FAK/PTK2 in cancer immunotherapy, we analyzed its correlations with tumor microenvironment and with immune processes/elements (e.g., immune cell infiltration, immunosuppressants, and stimulants) and major histocompatible complexes. Potential pathways associated with FAK/PTK2 signaling in cancers were also explored. Correlations between FAK/PTK2 and 2 immunotherapeutic biomarkers (tumor mutation load and microsatellite instability) were studied. Finally, the 3 independent immunotherapy cohorts were used to study the relationship between FAK/PTK2 and immunotherapeutic response. Although FAK/PTK2 is not closely associated with age (13/33), gender (5/33), or tumor stage (5/33) in any of the studied human cancers, it has potential prognostic value for predicting patient survival. Consistency between FAK/PTK2 activity and expression exists in some cancers (3/33). Generally, FAK/PTK2 is robustly correlated with immune cell infiltration, immune modulators, and immunotherapeutic markers. Moreover, high FAK/PTK2 expression is significantly related to immune-relevant pathways. However, FAK/PTK2 is not significantly correlated with the immunotherapeutic response. Research on the immunotherapeutic value of FAK/PTK2 in 33 human cancers provides evidence regarding the function of FAK/PTK2 and its role in clinical treatment. However, given the use of a bioinformatics approach, our results are preliminary and require further validation.

## 1. Introduction

Although immune checkpoint inhibitors (ICI) targeting programmed cell death protein 1, its ligand 1 or cytotoxic T lymphocyte antigen 4 significantly prolong the overall survival of patients with different types of cancers, only a limited subset of patients are beneficiary.^[1]^ Therefore, finding out reasonable and effective strategies to improve the efficacy of ICIs in cancerous patients is urgent. Understanding the genomic relevance of the response of ICIs will help to develop new biomarkers and therapies to enhance clinical response and expand the beneficiary population.^[2,3]^

Focal adhesions are important mediators in the interaction between the cytoskeleton and intracellular signaling molecules into the extracellular matrix through transmembrane receptors.^[4,5]^ This communication is mediated by the nonreceptor cytoplasmic protein tyrosine kinase focal adhesion tyrosine kinase (FAK/PTK2).^[6]^ This FAK/PTK2, located at the tip of chromosome 8q24.3,^[7]^ is defined as the phosphorylated substrate of viral Src oncogene and anchored at the integrin-rich cell adhesion site, which was first discovered by Hanks et al^[8]^ and Schaller et al^[9]^ in the early 1990s. Initially, Weiner et al^[10]^ found that FAK/PTK2 may be related to tumors. Moreover, FAK/PTK2 is overexpressed and activated in many types of advanced solid cancers, including colorectal,^[11]^ ovarian,^[12]^ esophageal,^[13]^ and hepatocellular^[14]^ carcinomas, and is associated with poor overall survival.^[7,15]^ FAK/PTK2 reportedly plays an important role in tumor adhesion, diffusion, motility, invasion, metastasis, survival, angiogenesis, epithelial to mesenchymal transformation, cancer stem cells, and the tumor microenvironment (TME).^[16–18]^

The TME consists of various immune cells, mesenchymal-origin cells, and extracellular matrix,^[19]^ which influence tumorigenesis at all stages by directly interacting with tumor cells.^[20]^ FAK/PTK2 promotes antitumor immune evasion by increasing the count of regulatory T cells in tumors.^[21]^ In myeloid-specific FAK/PTK2 knock-out mice, FAK/PTK2 can reduce the killing ability of neutrophils against pathogens.^[22]^ However, after FAK/PTK2 was silenced in tumor cells or became less metastasitic through FAK/PTK2 pharmacological inhibition,^[23,24]^ hematopoietic FAK/PTK2 in mice lacked a metastasis-promoting microenvironment.^[25]^ Also, FAK/PTK2 is pivotal in macrophage invasion and movement.^[26]^ These reports suggest that matrix FAK/PTK2 plays a regulatory role in tumor growth.

The advent of next-generation sequencing and large-scale genomics has shifted oncology research from single-gene analysis to pan-cancer analysis. The Cancer Genome Atlas (TCGA) based on epigenome, genomic, proteome, and transcriptome data from different cancers can be used to identify similarities and differences in important bioprocesses among different cancers.^[27]^ Therefore, with the large-scale RNA sequencing data from TCGA, we performed a pan-cancer genome analysis of FAK/PTK2 from 33 cancer types. This study was aimed to evaluate the expression of FAK/PTK2 in different cancer types; its prognostic value in different tumors; its relationship with tumor immune characteristics, and its association with drug response. Specifically, the tumor immune characteristics include intraturmoral immune invasion, checkpoint markers, tumor mutation load (TMB), and microsatellite instability (MSI), which have been identified as potential biomarkers for predicting therapeutic response with ICIs.

## 2. Materials and methods

### 2.1. Data collection and statistical processing

The genomic and clinicopathological information of 33 cancers was obtained from TCGA (https://portal.gdc.cancer.gov/) and the University of California Santa Cruz Xena Explorer (cohort: TCGA pan-cancer). Somatic mutation data were acquired from TCGA. For the therapeutic cohort, a systematic search was performed to identify the immune checkpoint blockade cohorts, which can be publicly retrieved and reported with complete clinical information. Three immunotherapeutic cohorts were finally employed: advanced urothelial cancer with atezolizumab intervention (IMvigor210 cohort from Mariathasan et al^[28]^), metastatic melanoma with pembrolizumab treatment (GSE78220 cohort from the Gene Expression Omnibus), and renal cell carcinoma with nivolumab treatment (GSE67501cohort from Gene Expression Omnibus). Statistical Analysis R software (version 4.1.2) was used for all statistical analysis and graphical visualization. Wilcoxon test was performed based on the FAK/PTK2 expression of nonresponder and responder groups. The Student *t* test or 1-way one-way analysis of variance test was used to compare 2 or more groups of continuous variables with a normal distribution (including expression and activation of FAK/PTK2 in 33 cancers).

### 2.2. Clinical correlation between FAK/PTK2 expression and various cancers

With package Limma in R Studio, differential gene expressions were analyzed to determine whether expression varied between tumor and normal groups. The correlation between FAK/PTK2 expression and other clinical parameters (age, gender, and tumor stage) was also investigated. To explore the time-dependent prognostic value of FAK/PTK2 in 33 cancers, we performed univariate Cox regression analysis on package Survival in R. The studied survival outcomes included overall survival (OS; period from the start of treatment to death from any cause), disease-free survival (DFS; period from the start of treatment to recurrence or death from any cause), disease-specific survival (DSS: cancer survival in the absence of other causes of death), and progression-free survival (PFS: period from the start of treatment to progression or death from any cause). The hazard ratio >1 indicates that the exposure factor FAK/PTK2 expression is a promoting factor of positive events (death). Variations with *P* < .05 are considered significant.

### 2.3. Gene set enrichment analyses

Gene set enrichment analyses (GSEA) provide a robust way to analyze molecular profiling data. To further investigate the protein level of FAK/PTK2 in pan-cancers, we used 69 relevant genes that were significantly up-regulated after 3-MC agonist treatment and down-regulated after GNF-351 antagonist treatment in 8 cell lines.^[29]^ FAK/PTK2 activity was detected by single-sample GSEA. After that, the difference in FAK/PTK2 activity between the normal and tumor groups was investigated. Then the mean values of expression and activity were calculated and arranged in 33 types of cancers, aiming to explore the potential features of FAK/PTK2 expression and activity.

### 2.4. Potential association between FAK/PTK2 expression and immune-related factors

The stromal score and immune score of each case were first calculated using the Estimation of Stromal and Immune cells in Malignant Tumour tissues using Expression data package, a tool for predicting tumor purity and the presence of infiltrating stromal/immune cells in tumor tissues.^[30]^ The Estimation of Stromal and Immune cells in Malignant Tumour tissues using Expression data is based on single-sample GSEA and generates 3 final scores: the stromal score (indicating the presence of stromal cells in tumor tissues), the immune score (representing the infiltration of immune cells in tumor tissues), and tumor purity. Then the abundance of immune cell infiltration in low FAK/PTK2-expressing and high FAK/PTK2-expressing groups was estimated using CIBERSORT, a deconvolution algorithm that evaluates the proportions of 22 tumor-infiltrating lymphocyte subsets.^[31]^ In short, the number of permutations was set to be 1000 and the samples with *P* < .05 in the cohort were eligible for further investigation. Correlations of FAK/PTK2 expression with TMB and MSI were also investigated, since these 2 indicators are closely associated with the immune response. TMB was defined as the total number of errors in somatic gene coding, base substitution, gene insertions, or deletions detected in every million bases. To calculate the TMB of each case, the total number of mutations counted was divided by the exome size (38 Mb). The MSI score of each TCGA cancer case was obtained from a previous study.^[32]^ In addition, the underlying relationship between FAK/PTK2 expression and immunological modulators (immune inhibitors, immune stimulators, and MHC molecules) was explored via TISIDB (http://cis.hku.hk/TISIDB/index.php). The 4 most relevant results were then highlighted and presented in plots. Finally, to further investigate the relevant signaling pathways, we performed GSEA to identify differential pathways between the low FAK/PTK2-expressing and high FAK/PTK2-expressing groups, which were obtained from the Kyoto Encyclopedia of Genes and Genomes. The relevant signaling pathways were presented in plots if they fulfilled certain criterion (*P* < .05) and the pathways with the top 5 normalized enrichment scores were considered.

### 2.5. Analysis of immunotherapeutic response

As mentioned above, 3 relevant independent immunotherapeutic cohorts were included and analyzed. In general, immunotherapeutic approaches yielded 4 outcomes: complete response, partial response, progressive disease, and stable disease. Patients who achieved complete response or partial response were categorized as responders and compared to nonresponders, who showed signs of stable disease or progressive disease. Then the differences in FAK/PTK2 expression between the responders and nonresponders were identified by the Wilcoxon test.

## 3. Results

### 3.1. Clinical landscape of FAK/PTK2 expression in 33 cancers

**Figure 1.**
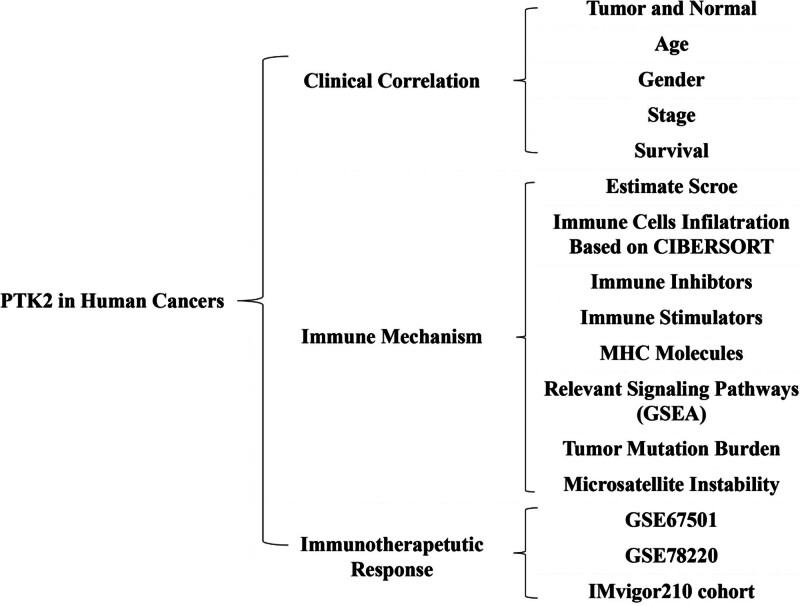
The analyses and indicators employed in our research. In clinical correlation section, differential FAK/PTK2 expression analyses were performed between different tissues (tumor vs normal), ages (≤65 vs >65), genders (male vs female), stages (stage I + II vs stage III + IV). Survival correlation analyses were based on univariate Cox regression analysis. In immune mechanism section, relevant signaling pathways were explored by GSEA based on the FAK/PTK2 expression. In immunotherapeutic response section, Wilcoxon test was performed based on the FAK/PTK2 expression of nonresponder and responder groups. FAK/PTK2 = focal adhesion tyrosine kinase, GSEA = gene set enrichment analyses.

**Figure 2.**
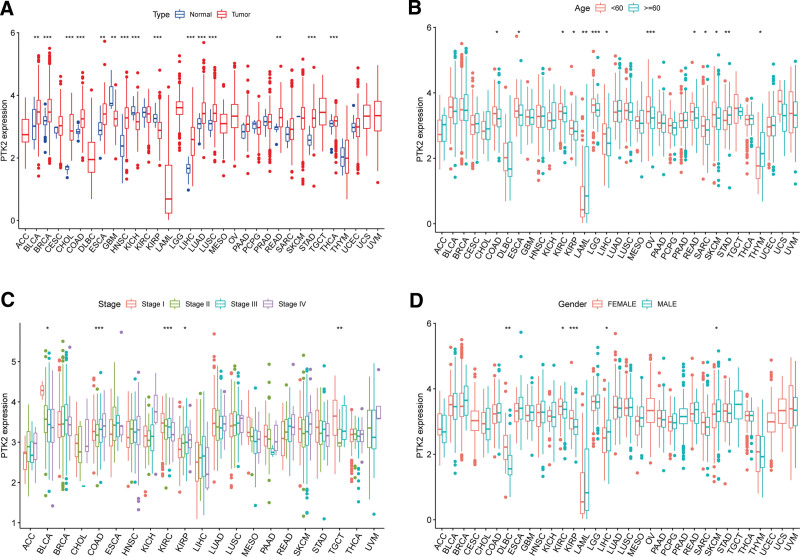
Paired test and one-way analysis of variance were used to study the expression of FAK/PTK2 in tumors and nontumors, different ages, different stages, and gender. (A) The differential expression analysis between tumor and normal groups of FAK/PTK2 in 33 cancers. (B) The correlation between age and FAK/PTK2. (C) The correlation between tumor stage and FAK/PTK2. (D) The correlation between gender and FAK/PTK2. *P < .05, **P < .01, ***P < .001. FAK/PTK2 = focal adhesion tyrosine kinase.

The analysis details are summarized and presented for a more comprehensive outlook Figure 1. The abbreviations and full names of the 33 cancers considered here are available in Table 1. FAK/PTK2 is differentially expressed in 15 of the 33 cancers (BLCA, BRCA, CHOL, COAD, ESCA, GBM, HNSC, KICH, KIRP, LIHC, LUAD, LUSC, RWAD, STAD, THCA) (Fig. 2A). FAK/PTK2 is highly differentially expressed among elder patients with LAML, STAD or THYM, whereas it is weakly expressed in COAD, ESCA KIRC, KIPR, LGG, MESO, PCPG, PRAD, and READ (Fig. 2B). Meanwhile, FAK/PTK2 expression is significantly correlated with tumor stage of some cancers, including BLCA, COAD, KIRC, KIRP, and TGCT (Fig. 2C). Besides, the results indicate significant gender-based differences in FAK/PTK2 expression of the DLBC, KIRC, KIRP, LIHC, and SKCM cases (Fig. 2D).

**Table 1:** Thirty-three types of human cancers employed in our research (abbreviation full name).

ACC	Adrenocortical carcinoma
BLCA	Bladder urothelial carcinoma
BRCA	Breast invasive carcinoma
CESC	Cervical squamous cell carcinoma and endocervical adenocarcinoma
CHOL	Cholangiocarcinoma
COAD	Colon adenocarcinoma
DLBC	Lymphoid neoplasm diffuse large B-cell lymphoma
ESCA	Esophageal carcinoma
GBM	Glioblastoma multiforme
HNSC	Head and neck squamous cell carcinoma
KICH	Kidney chromophobe
KIRC	Kidney renal clear cell carcinoma
KIRP	Kidney renal papillary cell carcinoma
LAML	Acute myeloid leukemia
LGG	Brain lower-grade glioma
LIHC	Liver hepatocellular carcinoma
LUAD	Lung adenocarcinoma
LUSC	Lung squamous cell carcinoma
MESO	Mesothelioma
OV	Ovarian serous cystadenocarcinoma
PAAD	Pancreatic adenocarcinoma
PCPG	Pheochromocytoma and paraganglioma
PRAD	Prostate adenocarcinoma
READ	Rectum adenocarcinoma
SARC	Sarcoma
SKCM	Skin cutaneous melanoma
STAD	Stomach adenocarcinoma
TGCT	Testicular germ cell tumors
THCA	Thyroid carcinoma
THYM	Thymoma
UCEC	Uterine corpus endometrial carcinoma
UCS	Uterine carcinosarcoma
UVM	Uveal melanoma

**Figure 3.**
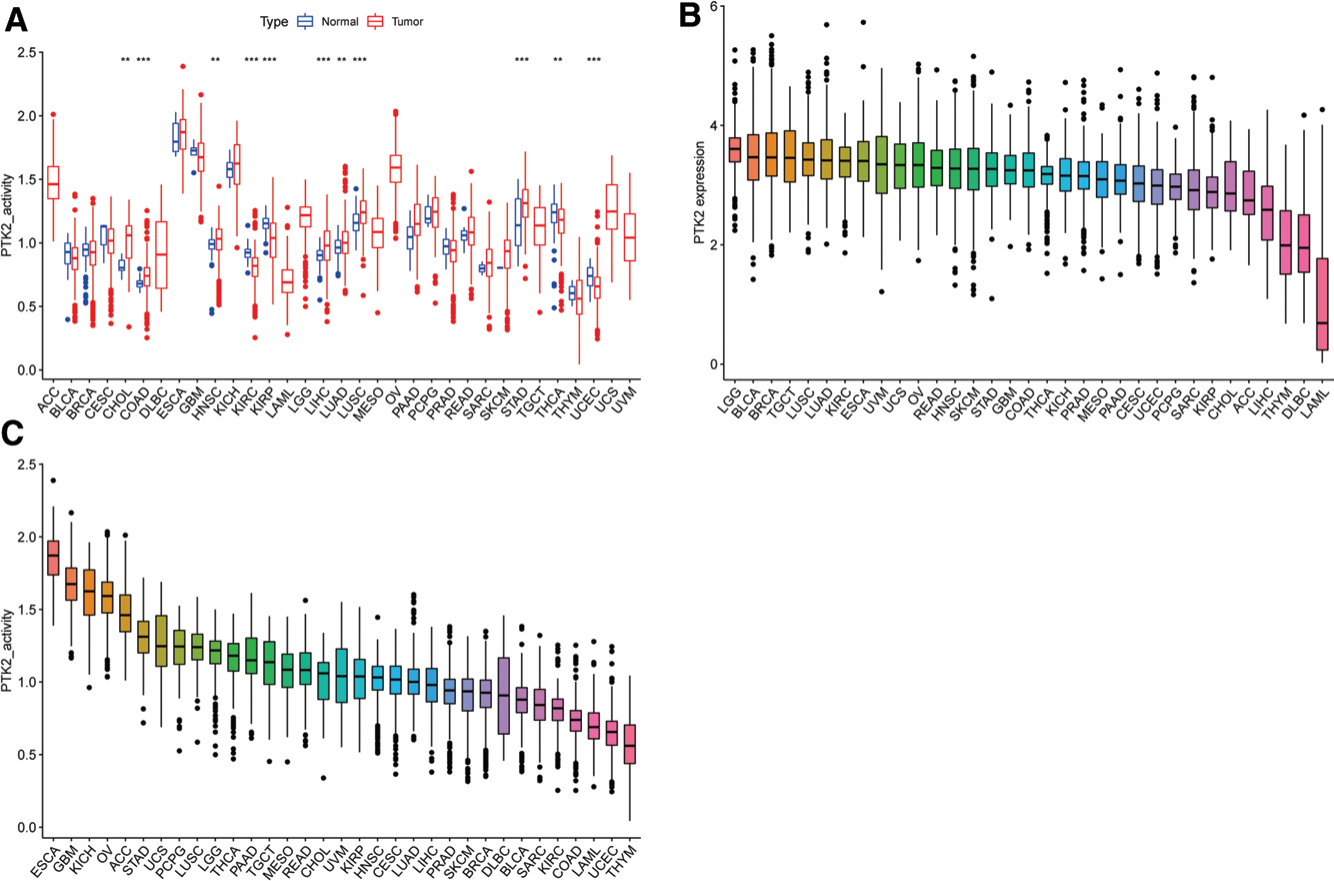
Generation and investigation of FAK/PTK2 activity. (A) The different activity analysis between tumor and normal groups of FAK/PTK2 in 33 cancers. (B) The mean expression of FAK/PTK2 in 33 cancers (from high to low). (C) The mean activity of FAK/PTK2 in 33 cancers (from high to low). **P < .01 and ***P < .001. FAK/PTK2 = focal adhesion tyrosine kinase.

FAK/PTK2 activity is significantly increased in tumor groups of CHOL, COAD, HNSC, LIHC, LUAD, LUSC, and STAD, and is decreased in tumor groups of KIRC, KIRP, THCA, and UCEC (Fig. 3A). FAK/PTK2 expression varies across tumors from high to low (Fig. 3B). The gene activity of FAK/PTK2 differs from high to low among different tumors (Fig. 3C).

**Figure 4.**
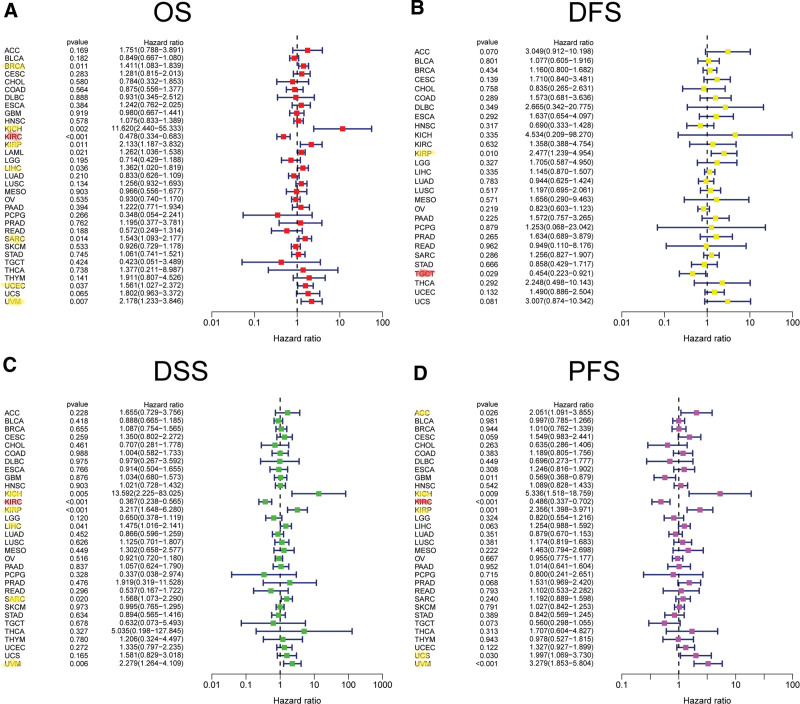
The forest plots of univariate Cox regression analyses. The highlighted items mean that FAK/PTK2 expression was significantly correlated with prognosis in these types of cancers (*P* < .05). Items with hazard ratio >1 indicated that the FAK/PTK2 expression was a promoting factor of death. (B) DFS = disease-free survival, (C) DSS = disease-specific survival, FAK/PTK2 = focal adhesion tyrosine kinase, (A) OS = overall survival, (D) PFS = progression-free survival.

According to the forest plots (Fig. 4A–D), the association between FAK/PTK2 expression and OS is apparently positive in BRCA, KICH, KIRP, LAML, LIHL, SAPC, UCEC, and UVM, but is negative in KIRC (Fig. 4A).

**Figure 5.**
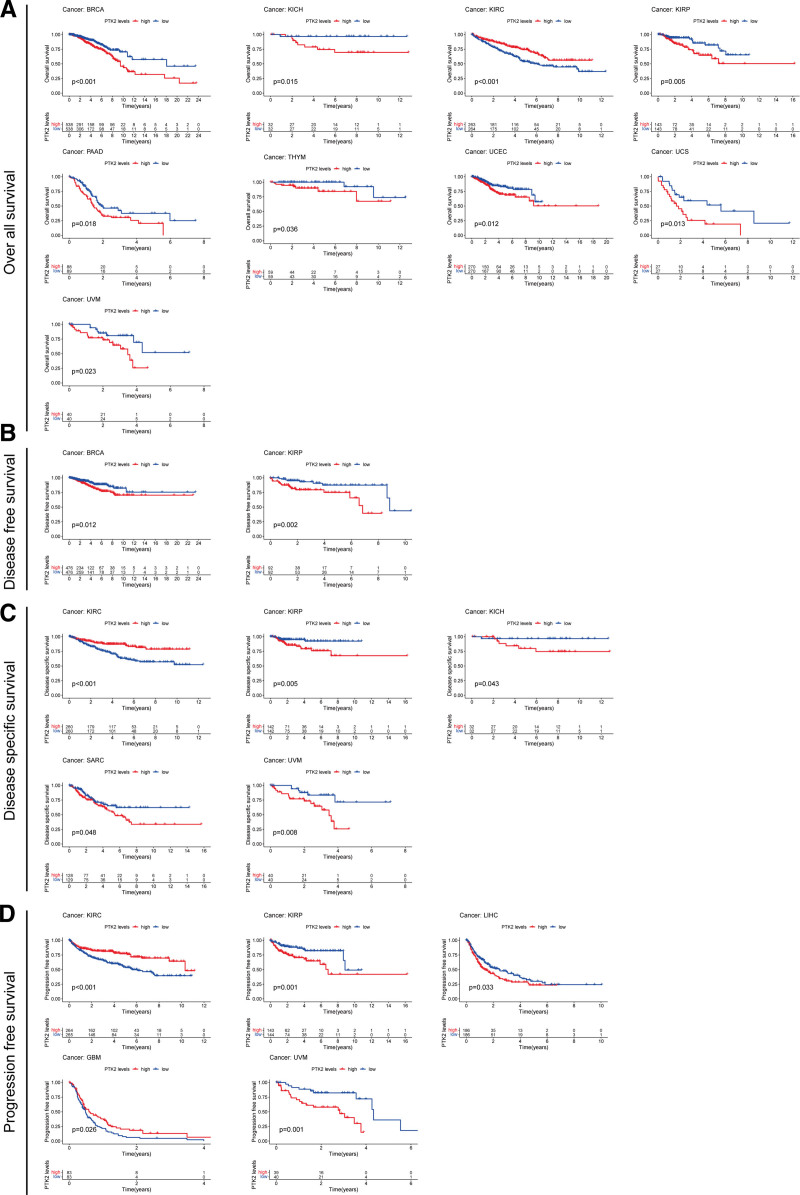
Univariate Cox regression analysis of the survival outcomes included (A) OS, (B) DFS, (C) DSS, and (D) PFS, effect of FAK/PTK2 expression on prognosis in different tumor species. DRS = disease-free survival, DSS = disease-specific survival, FAK/PTK2 = focal adhesion tyrosine kinase, OS = overall survival, PFS = progression-free survival.

As for FAK/PTK2 and DFS, a significant negative association exists in TGCT, but a positive association is present in KIRP (Fig. 4B). In terms of DSS, FAK/PTK2 expression has a protective effect on KIRC, whereas it seems to be a risk factor in KICH, KIRP, LIHC, SARC, and UVM (Fig. 4C). Moreover, the PFS forest plot confirms the protective role of FAK/PTK2 expression in GBM and KIRC, and its role as a risk factor in ACC, KICH, KIRP, UCS, and UVM (Fig. 4D). The effect of FAK/PTK2 on prognosis in different tumors is shown in Figure 5.

### 3.2. Underlying association between FAK/PTK2 expression and immune-related factors

**Figure 6.**
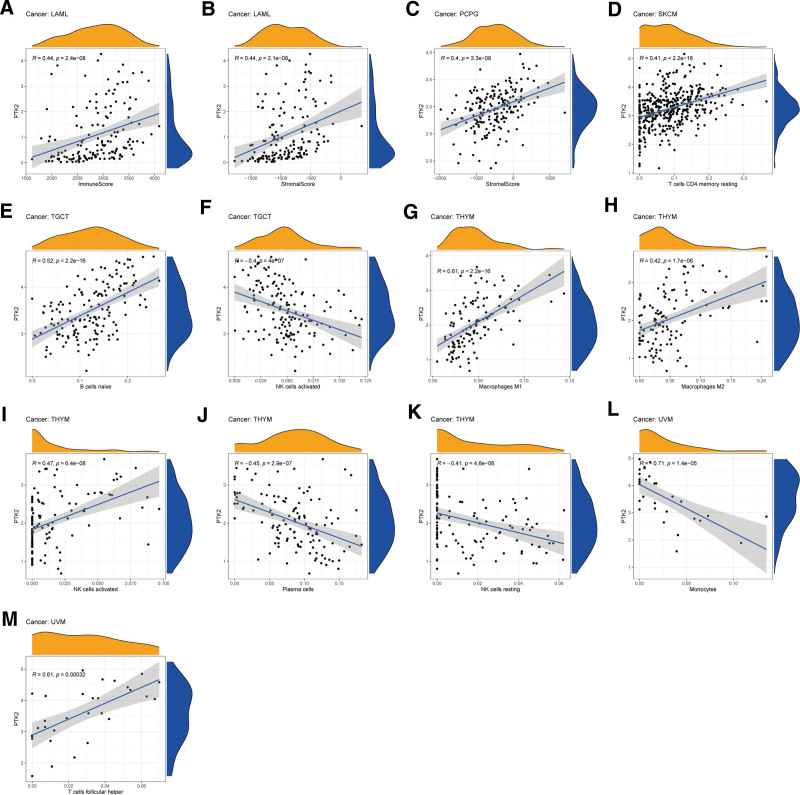
The correlation between FAK/PTK2 expression and both the ESTIMATE score and immune cell infiltration. The ESTIMATE score includes stromal score (indicates the presence of stromal cells in tumor tissues), immune score (represents the infiltration of immune cells in tumor tissues), and tumor purity. The immune cell infiltration was calculated by CIBERSORT algorithm. The correlation plots were illustrated if *R* > 0.5 and *P* < .05. ESTIMATE = Estimation of Stromal and Immune cells in Malignant Tumour tissues using Expression data, FAK/PTK2 = focal adhesion tyrosine kinase.

The stromal score, immune score, and immune cell infiltration are summarized in Figure 6 (*P* < .01 and |*R*| > 0.4). Notably, FAK/PTK2 expression is positively associated with the stromal scores of both LAML and PCPG, and with the immune score of LAML (all *R* < 0.5). In terms of immune cell infiltration, the FAK/PTK2 expression is positively associated with the M0/M1/M2 macrophage count in THYM, and with the count of NK cells activated, but R is over 0.5 only in M1 (*R* = 0.61, *P* < 2.2e−16). Besides, the FAK/PTK2 expression is negatively associated with the counts of plasma cells and NK cells resting. In UVM, FAK/PTK2 expression is associated negatively with monocyte infiltration (*R* = −0.71, *P* = 1.4e−0.5) and positively with T cell follicular helper infiltration (*R* = 0.61, *P* < .005) and the count of B cells naive (*R* = 0.52, *P* < 2.2e−16).

**Figure 7.**
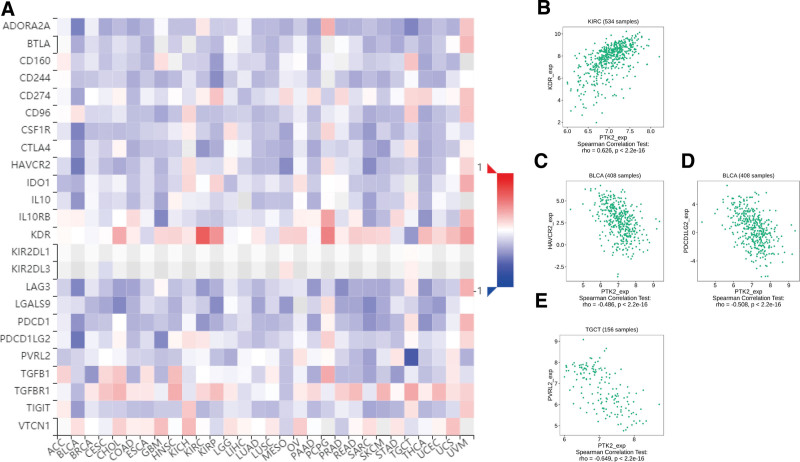
The expression correlation between FAK/PTK2 and immune inhibitors. Red indicates positive correlation whereas blue indicates negative correlation. The top 4 strongest associations were displayed via dotplots. FAK/PTK2 = focal adhesion tyrosine kinase.

**Figure 8.**
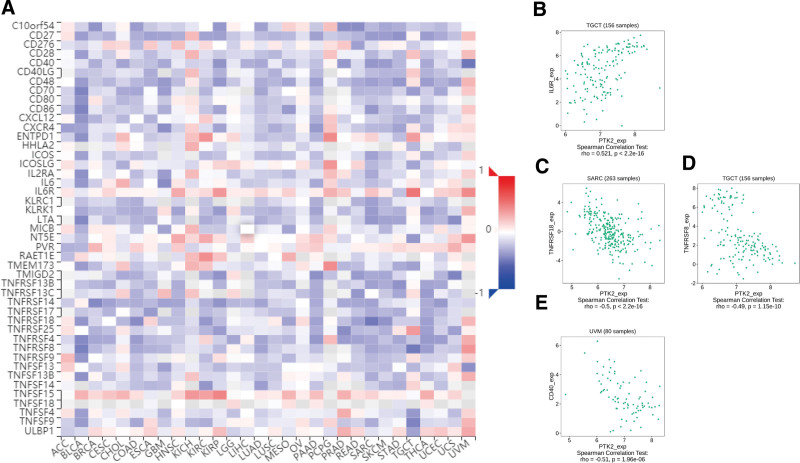
The expression correlation between FAK/PTK2 and immune stimulators. Red indicates positive correlation whereas blue indicates negative correlation. The top 4 strongest associations were displayed via dotplots. FAK/PTK2 = focal adhesion tyrosine kinase.

**Figure 9.**
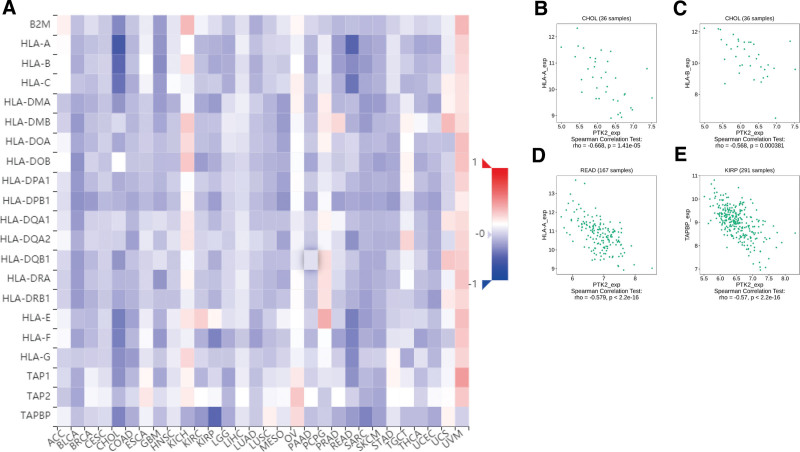
The expression correlation between FAK/PTK2 and MHC molecules. Red indicates positive correlation whereas blue indicates negative correlation. The top 4 strongest associations were displayed via dotplots. FAK/PTK2 = focal adhesion tyrosine kinase, MHC = major histocompatibility.

Totally 24 types of immune inhibitors were analyzed. FAK/PTK2 expression is associated positively with KDR in KIRC, and negatively with PVRL2 in TGCT, HAVCR2, and PDCD1LG2 in BLCA (Fig. 7). The correlation analyses of 45 immune stimulators demonstrate that FAK/PTK2 expression is associated positively with IL-6R in TGCT and negatively with TNFRSF18 in SARC, TNFRSF8 in TGTC, and CD40 in UVM (Fig. 8). Moreover, the expression correlation between FAK/PTK2 and MHC molecules demonstrate that FAK/PTK2 expression is negatively associated with HLA-A in CHOL, HLA-B in CHOL, HLA-A in READ, and TAPBP in KIRP (Fig. 9).

**Figure 10.**
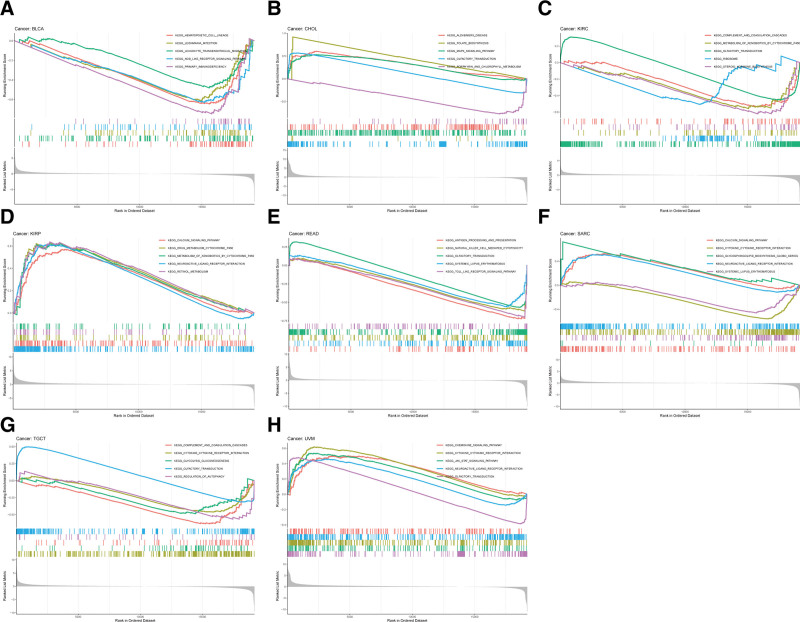
The results of GSEA based on KEGG database. In each panel, the pathways marked in the left were enriched in the high FAK/PTK2 expression group, while the pathways marked in the right were enriched in the low FAK/PTK2 expression group. FAK/PTK2 = focal adhesion tyrosine kinase, GSEA = gene set enrichment analyses, KEGG = kyoto encyclopedia of genes and genomes.

**Figure 11.**
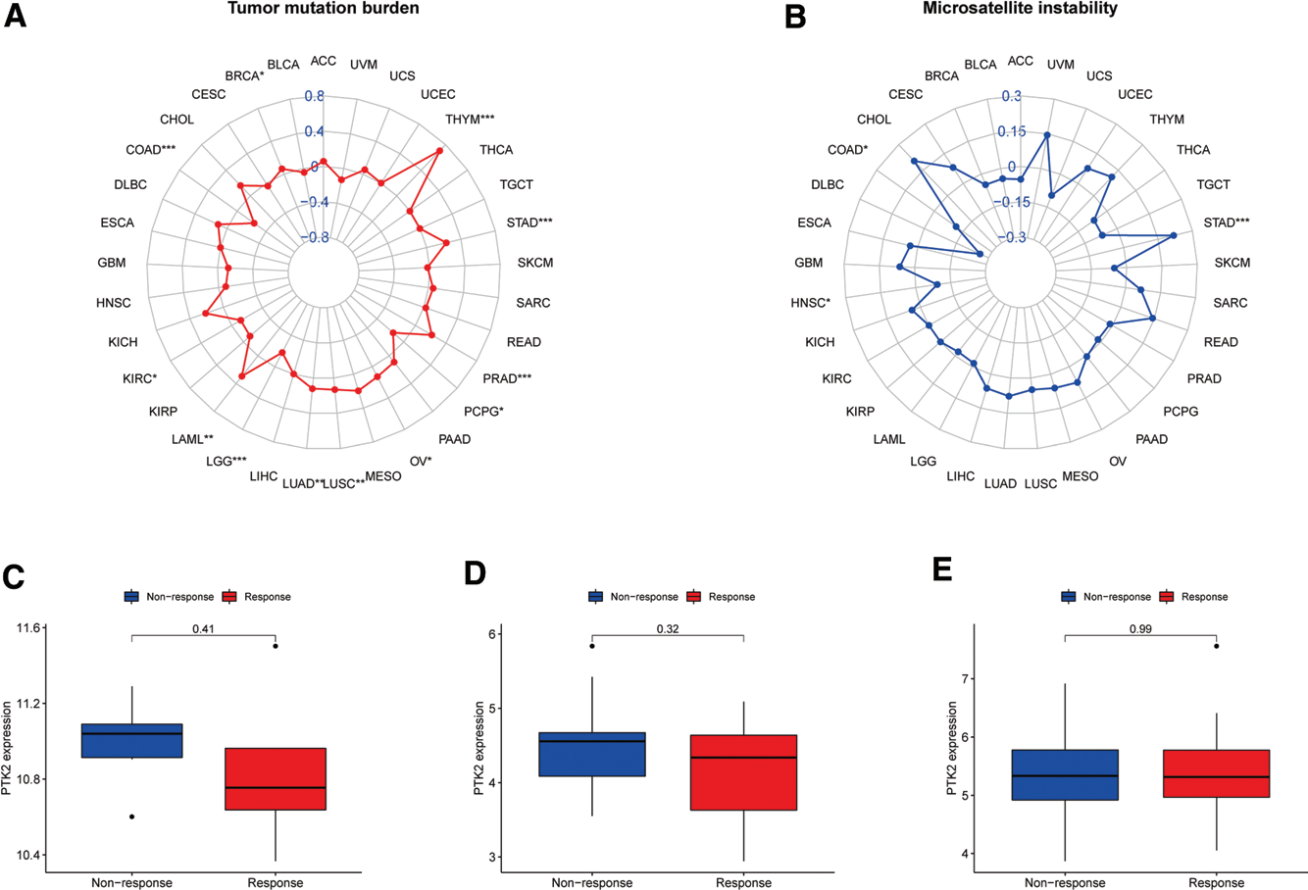
The correlation between FAK/PTK2 and both immunotherapeutic markers and the immunotherapeutic response. *P < .05, **P < .01, and ***P < .001. FAK/PTK2 expression is related to TMB positively in THYM, LAML, LUAD, LUSC, OV, PRAD, STAD, and BRCA, but negatively in COAD, PCPG, LGG, and KIRC (A), MSI is associated with FAK/PTK2 expression positively in STAD and negatively in COAD and HNSC (B). No significant difference in FAK/PTK2 expression is evident between the responders and nonresponders in any of the 3 independent cohorts (C–E). FAK/PTK2 = focal adhesion tyrosine kinase.

Given the robust correlations of FAK/PTK2 with TGCT, KIRC, CHOL, KIRP, READ, UVM, BLCA, and SARC, we performed GSEA to investigate the potential pathways involved in FAK/PTK2 signaling in these cancers. Genes from immune-relevant pathways (e.g., the drug metabolism cytochrome P450 signaling pathway) tend to be enriched in the high expressing groups of PRAD, but the Cytokine receptor interaction signaling pathway is enriched in the high expressing groups of UVM (Fig. 10). The correlation between FAK/PTK2 and the novel dynamic biomarkers of the immune checkpoint blockade (TMB and MSI) were further explored. FAK/PTK2 expression is related to TMB positively in THYM, LAML, LUAD, LUSC, OV, PRAD, STAD, and BRCA, but negatively in COAD, PCPG, LGG, and KIRC (Fig. 11A). MSI is associated with FAK/PTK2 expression positively in STAD and negatively in COAD and HNSC (Fig. 11B).

### 3.3. Immunotherapeutic response of FAK/PTK2

No significant difference in FAK/PTK2 expression is evident between the responders and nonresponders in any of the 3 independent cohorts (Fig. 11C–E). A trend observed in the studied cohorts is that patients with low FAK/PTK2 expression are seemingly more responsive to immunotherapy.

## 4. Discussion

This study extracted potential values of FAK/PTK2 in various cancers, especially in the context of immunotherapy. First, correlation analysis between FAK/PTK2 and clinical parameters shows no significant differences in age, gender, or tumor stage among most cancer types. FAK/PTK2 is overexpressed in BLCA, BRCA, CHOL, COAD, ESCA, GBM, HNSC, KICH, KIRP, LIHC, LUAD, LUSC, RWAD, STAD, and THCA, which is consistent with previous studies that FAK/PTK2 expressions in HNSC,^[33]^ BRCA, and LIHC^[34]^ are significantly higher than in the surrounding normal tissues. In HNSC, strong FAK/PTK2 expression is robustly associated with an increased HNSC risk and more importantly, FAK/PTK2 expression is an independent predictor in multivariate analysis.^[35]^ Moreover, FAK/PTK2 overexpression is associated with local infiltration and distant metastasis.

To further clarify the action mechanism of FAK2 overexpression in tumor growth, in vitro experiments from Ilic et al^[36]^ reveal that endothelial cells lacking FAK/PTK2 expression or function have a severely reduced ability to form tubules in the stromal gel. The global FAK/PTK2 knock in point mutation in the catalytic domain by Lim et al^[37]^ further confirms the role of FAK/PTK2 in the development process, and reveals that the activity of FAK/PTK2 is very important for vascular morphogenesis cell movement and polarity. Furthermore, PTK2 serves as a target for 8q23-q24 amplification events in LIHC, and elevated PTK2 expression is correlated with large tumor volume.^[34]^ FAK/PTK2 can also be translocated to the nucleus of cancer cells, where it regulates the expression program of chemokines and cytokines in inflammatory genes,^[38]^ so as to promote immune escape and immune therapy resistance.^[39,40]^ Besides, the expression and tyrosine phosphorylation of FAK are highly correlated with cell cycle progression by modulating cell cycle-relative molecules, which highlights that FAK functions as a key regulator in promoting cancer proliferation.^[41]^ Tai et al^[42]^ confirm that FAK activation, as determined by phosphospecific antibody recognition of the FAK tyrosine autophosphorylation site, is increased with tumor progression. Hence, the above evidences confirm the usefulness of FAK/PTK2 in cancer prognosis. In a word, these data suggest that FAK/PTK2 is a regulator of tissue growth response and malignant transformation.

To clarify the relationship between FAK/PTK2 overexpression and prognosis, we demonstrate that high FAK/PTK2 expression is associated with worse OS in BRCA, KICH, KIRP, PAAD, THYM, UCEC, UCS, and UVM, worse DFS in BRCA, KIRP, and KICH, worse DSS in KIRP, SARC, and UVM, and worse PFS in KIRP, LIHC, and UVM. These results are consistent with multiple previous studies. For instance, Golubovskaya et al^[43]^ confirm that high FAK expression always predicts poor prognosis in BRCA. Miyazaki et al^[13]^ suggest that FAK is highly expressed in esophageal cancer and is closely associated with poor prognosis. We hypothesize that therapeutic modulation of FAK/PTK2 activity or phosphorylation level in various tumors may be an effective strategy with clinical benefits.

We further investigated the correlation between FAK/PTK2 and immune cell infiltration and observed a robust positive correlation between FAK/PTK2 and M0/M1/M2 macrophages in THYM. Reportedly, FAK/PTK2 affects tumor development as well as immune responses within the tumor environment via tumor-associated macrophages.^[44]^ FAK is important to induce TAMs by recruiting macrophages into tumor tissues, and may perform pretumor function by regulating the expression of downstream genes.^[42]^ T cell infiltration is a reliable predictor of prognosis and has been implemented in the treatment of various cancers.^[45]^ Reportedly, T cells play a positive role in tumor progression and their exclusion from TME leads to immune privilege.^[46]^ In UVM, FAK/PTK2 expression is associated negatively with monocyte infiltration (*R* = −0.71, *P* = 1.4e−0.5) and positively with T cell follicular helper infiltration (*R* = 0.61, *P* < .005) and B-cell naive count (*R* = 0.52, *P* < 2.2e−16). Similarly, previous research shows that the prognosis of UVM is inversely associated with immune cell infiltration.^[47]^

In addition, 2 immunotherapeutic biomarkers (TMB and MSI) are significantly correlated with FAK/PTK2 in some cancers. Generally, more new antigens may be formed in a tumor with more somatic mutations, and TMB provides a useful tumor neoantigen load.^[48]^ In contrast, MSI is defined as a robust mutant phenotype caused by DNA mismatch repair defects and is a potential predictive marker of immunotherapy.^[49]^ FAK/PTK2 is correlated negatively with TMB and MSI in COAD, but positively with these 2 biomarkers in STAD. It is suggested that FAK/PTK2 may indirectly affect the immunotherapeutic response of STAD and COAD. Subsequently, we studied the correlation between FAK/PTK2 and immunotherapeutic response, but found no significant differences between the 2 study cohorts. We hypothesize that although these 3 cohorts have received anti-death protein 1 treatment and responded, FAK/PTK2 may affect the immunotherapeutic response by targeting other immune checkpoints, such as cytotoxic T lymphocyte antigen 4 or TIGIT. Moreover, we only analyzed 3 related cohorts and can hardly clarify the actual immunotherapeutic response of FAK/PTK2. Hence, more relevant immunotherapy cohorts shall be studied in the future.

To our knowledge, this is the first study to focus on the value of FAK/PTK2 in various cancers (33 types). This study provides valuable insights into the role of FAK/PTK2 in cancer immunotherapy, reveals its relationship with important immune indicators (immune cell infiltration, immune modulators, and immune biomarkers), and may help to understand the potential mechanism between FAK/PTK2 and the immune system. Although not all cancers show an association between tumor immune microenvironment and FAK/PTK2, these findings highlight the immune role of FAK/PTK2 in specific cancers, which will be used as an effective means to target them. However, given the bioinformatics method used here, these findings are preliminary. Therefore, more research on this topic is needed before the association between FAK/PTK2 and cancer immunotherapy is clearly understood and widely accepted.

There are still some limitations in the current study. First of all, this study showed that patients with low FAK/PTK2 expression are more responsive to immunotherapy, but the target molecules of FAK/PTK2 to regulate the immunotherapeutic response of cancer is unknown. Second, We did not conduct clinical trials to further validate our findings, we should go back to the clinical samples to confirm the key conclusions so the results still need further verification.

## 5. Conclusions

To our knowledge, this is the first study to present a high frequency of FAK/PTK2 expression and the predictive significance of ICI treatment in 33 human cancers. We believe that these findings may lay a groundwork for prospective functional experiments and may eventually have an impact in the clinical setting.

## Author contributions

**Conceptualization:** Yujing Shi, Yumeng Zhang, Liang Liang.

**Data curation:** Yujing Shi, Yumeng Zhang, Xiaoke Di.

**Formal analysis:** Yujing Shi, Xiaoke Di.

**Investigation:** Yujing Shi, Xiaoke Di.

**Methodology:** Yujing Shi, Mengyang Ju, Yumeng Zhang.

**Writing—original draft:** Yujing Shi, Xiaoke Di.

**Software:** Mengyang Ju, Xinchen Sun.

**Supervision:** Liang Liang, Xinchen Sun.

**Validation:** Liang Liang, Xinchen Sun, Xiaoke Di.

**Writing—review & editing:** Xinchen Sun, Xiaoke Di.

**Funding acquisition:** Xiaoke Di.

**Visualization:** Xiaoke Di.
